# Transplantation of Human Glial Progenitors to Immunodeficient Neonatal Mice with Amyotrophic Lateral Sclerosis (SOD1/rag2)

**DOI:** 10.3390/antiox11061050

**Published:** 2022-05-26

**Authors:** Luiza Stanaszek, Piotr Rogujski, Katarzyna Drela, Michal Fiedorowicz, Piotr Walczak, Barbara Lukomska, Miroslaw Janowski

**Affiliations:** 1 NeuroRepair Department, Mossakowski Medical Research Institute, Polish Academy of Sciences, 02-106 Warsaw, Poland; lstanaszek@imdik.pan.pl (L.S.); progujski@imdik.pan.pl (P.R.); barbara.lukomska@imdik.pan.pl (B.L.); 2 Medical Research Agency, 00-014 Warsaw, Poland; katarzyna.drela@abm.gov.pl; 3 Small Animal Magnetic Resonance Imaging Laboratory, Mossakowski Medical Research Institute, Polish Academy of Sciences, 02-106 Warsaw, Poland; mfiedorowicz@imdik.pan.pl; 4 Center for Advanced Imaging Research, Department of Diagnostic Radiology and Nuclear Medicine, University of Maryland Marlene and Stewart Greenebaum Comprehensive Cancer Center, University of Maryland, Baltimore, MD 21201, USA; pwalczak@som.umaryland.edu

**Keywords:** GRPs, ALS, immunodeficient, mice, MRI, neurological disorders

## Abstract

Amyotrophic lateral sclerosis (ALS) is a progressive, fatal disease with no effective therapy. The neurodegenerative character of ALS was an appealing target for stem cell-based regenerative approaches. Different types of stem cells have been transplanted in both preclinical and clinical settings, but no convincing outcomes have been noted. Human glial restricted precursors (hGRPs) transplanted intraventricularly to neonatal, immunodeficient mice rescued lifespan of dysmyelinated mice. Intraspinal injection of hGRPs also provided benefits in the mouse model of ALS. Therefore, we have recently developed an immunodeficient model of ALS (double mutant SOD1/rag2), and, in this study, we tested the strategy previously used in dysmyelinated mice of intraventricular transplantation of hGRPs to immunodeficient mice. To maximize potential therapeutic benefits, the cells were implanted into neonates. We used magnetic resonance imaging to investigate the progression of neurodegeneration and therapeutic responses. A cohort of animals was devoted to survival assessment. Postmortem analysis included immunohistochemistry, Nissl staining, and Western blots. Cell transplantation was not associated with improved animal survival, slowing neurodegeneration, or accumulation of misfolded superoxide dismutase 1. Postmortem analysis did not reveal any surviving hGRPs. Grafting into neonatal immunodeficient recipients did not prevent ALS-induced cell loss, which might explain the lack of positive therapeutic effects. The results of this study are in line with the modest effects of clinical neurotransplantations. Therefore, we urge stem cell and ALS communities to develop and implement cell tracking methods to better understand cell fates in the clinic.

## 1. Introduction

Amyotrophic lateral sclerosis (ALS) is a progressive, fatal disease in which most patients die within three to five years of diagnosis. While we are witnessing an unprecedented explosion of basic and clinical research, thus far, no therapy offers a significant clinical benefit for patients with ALS [1]. Therefore, many therapeutic approaches are being explored in the context of ALS.

The detailed etiopathogenesis of ALS is still elusive, although the accumulation of misfolded superoxide dismutase 1 (SOD1) protein in neurons seems to be a critical downstream pathology. It has been recently shown that the accumulation of misfolded SOD1 correlates with corticospinal and spinal motor neuron degeneration [2]. Moreover, it was revealed that overexpression of wild-type SOD1 also leads to its misfolding [3]. Misfolded SOD1 has also been found in the cerebrospinal fluid (CSF) of patients with sporadic ALS [4]. In a very interesting study, it has been reported that endoplasmic reticulum stress leads to the accumulation of wild-type SOD1 aggregates associated with sporadic amyotrophic lateral sclerosis [5]. Therefore, the prevention or containment of SOD1 misfolding and accumulation might be a universal strategy to tackle ALS.

Cell therapies for ALS have been attempted for decades [6], with various scenarios investigated. Mesenchymal stem cells were shown to be beneficial because of their immunomodulatory properties [7,8,9]. The toxic effect of ALS-derived astrocytes on motor neurons has also been demonstrated [7,10]. This further fueled the non-cell-autonomous hypothesis of degeneration, providing a rationale for targeting glia. Indeed, it was shown that reduction in SOD1 expression in astrocytes results in a delay of disease progression or onset of the disease as well as reduced microglia activation [11]. Moreover, it was proven that the wild-type, healthy environment of non-neuronal cells can diminish the degenerative changes in the spinal cord even if motoneurons (MNs) express mutated SOD1 [12]. In turn, the other type of glia—oligodendrocytes—except for involvement in myelinating processes, are responsible for providing neurons with energy in the form of lactate through monocarboxylate transporter (MCT-1). It was shown that in mouse and canine models of ALS, as well as in patients, the levels of MCT-1 are reduced, thus indicating the role of oligodendrocytes in ALS pathology by disrupting the metabolic support of neurons [13,14]. It has also been shown that glial-restricted progenitors (GRPs) allotransplanted to the cervical spinal cord extended the lifespan of ALS rats, but no cure was achieved [15].

Therefore, both macroglial phenotypes are attractive therapeutic targets. We have previously shown that highly migratory human glial restricted precursors (hGRPs) can replace abnormal mouse host glia and cure mice suffering from lethal dysmyelination. However, mouse GRPs (mGRPs) were characterized by a lower migratory potential and a lack of positive therapeutic effects [16]. In turn, our recent results with transplantation of canine GRPs in the same dysmyelinated mouse proved the functionality of transplanted cells in terms of myelination, however, with only partial effect in survival prolongation (30%) [17]. These findings are well-aligned with our previous studies, which indicate limited intraparenchymal migration of bone marrow- or cord blood-derived cells as the main driver of narrowing their therapeutic effects [18,19]. A predominant strategy used in previous studies was cell transplantation into symptomatic animals with a relatively short period of expected survival, which could be insufficient for stem and progenitor cells to differentiate and integrate within the host tissue. Immunological discordance between graft and host in most studies could be another source of failure. Therefore, we have developed immunodeficient ALS mice with lower SOD1 copy numbers capable of extended survival [20]. To further facilitate positive therapeutic effects, as we previously reported in dysmyelinating disease [16,17], we decided to transplant ALS mice at the neonatal stage. Early grafting also maximizes the time for cells to integrate and demonstrate therapeutic effects. While the spinal cord is a natural target for implantation, the size of the neonatal spinal cord is too small for direct cell transplantation. Therefore, we decided on the intracerebroventricular delivery, especially as with this route, we have previously observed cell migration throughout the CNS of dysmyelinated mice, including the spinal cord [16].

## 2. Materials and Methods

### 2.1. hGRPs

Human glial progenitors were obtained from the mid-gestation fetal brain and sorted on a magnetic-activated cell sorter (MACS). Cells on the third passage (Q-cells^®^) were kindly provided by Q Therapeutics and used for transplantation directly upon thawing [21].

### 2.2. Immunocytochemistry

The poly-L-lysine (Sigma-Aldrich, St. Louis, MO, USA) and laminin (Thermo Fisher Scientific, Waltham, MA, USA)-coated coverslips were dropped into the 24-well plate, and Q-cells^®^ (4th passage) were seeded. After 1, 5, and 9 days in culture cells were fixed with 4% PFA for 20 min and washed 3 times in PBS. Blocking solution (10% NGS, 5% BSA, 0.25% Triton X-100) was applied for 1 h, RT. Primary antibodies were used to distinguish cell phenotype: A2B5 (1:200) (MAB312R, Merck KGaA, Darmstadt, Germany) and NG2 (1:200) (AB5320, Merck KGaA, Darmstadt, Germany) for glial progenitors, Olig2 (1:500) (ABN899, Merck KGaA, Darmstadt, Germany) for oligodendrocyte precursors, MBP (1:200) (MAB386, Merck KGaA, Darmstadt, Germany), and GalC (1:200) (AB142, Merck KGaA, Darmstadt, Germany) for mature oligodendrocytes. After washing in PBS, secondary antibodies conjugated to Alexa fluorochromes were used: goat anti-rat 546, goat anti-rabbit 546, goat anti-rabbit 488, and goat anti-mouse 488 (all at 1:500 dilutions, Thermo Fisher Scientific, Waltham, MA, USA). Nuclei were counterstained with 5 μM Hoechst 33258 (Thermo Fisher Scientific, Waltham, MA, USA). Cells were imaged using a confocal laser scanning microscope (LSM 780, Carl Zeiss, Jena, Germany) and Cell Observer SD (Carl Zeiss, Jena, Germany) using 10× or 20× objectives with a tile scan command. Three samples were photographed per antibody.

### 2.3. RT-qPCR

Real-time quantitative polymerase chain reaction (RT-qPCR) was used to evaluate the expression level of selected molecules over the consecutive Q-Cell^®^ passages: fourth, fifth, and sixth. We have chosen for analysis three antigens representing subsequent steps of differentiation: glial progenitors (NG2) and oligodendrocyte precursors (Olig2). We have also tested the expression of the four neurotrophic factors: TGFβ, GDNF, EGF, and BDNF. Briefly, the Chomczyński method was used for mRNA isolation from cells [22]. Briefly, cells were collected, resuspended, and homogenized in TRIzol reagent and incubated at room temperature for 10 min. Next, homogenates were centrifuged at 12,000× *g* for 15 min at 4 °C. Aqueous phases were collected, precipitated with isopropyl alcohol, and incubated at room temperature for 10 min. Next, samples were centrifuged at 12,000× *g* for 15 min at 4 °C. Supernatants were discarded, pellets were washed with 75% ethanol, mixed, and centrifuged at 7500× *g* for 5 min. RNA pellets were air-dried at room temperature for 10 min and resuspended in DEPC-treated water. Reverse transcription was performed using a high-capacity cDNA reverse transcription kit (Thermo Fisher Scientific, Waltham, MA, USA) according to manufacturer protocol. RT-qPCR was made with the use of primers listed below in Table 1 and the SYBR green PCR master mix (Thermo Fisher Scientific, Waltham, MA, USA). The reaction was performed in 20 µL of the final volume in standard RT-qPCR conditions, using a 7500 Applied Biosystems system (Thermo Fisher Scientific, Waltham, MA, USA). GAPDH was used as an internal control. The relative protein expression level was calculated by the delta–delta CT method, where the first day of in vitro culture (passage third) was treated as a control.

### 2.4. SOD1/rag2 Mice

All animal procedures were performed following the protocol approved by the Warsaw Ethical Committee (IV Local Committee in Warsaw, 48/2013, 240/2017, 259/2017). Mice were bred and genotyped as described before [20]. Immunocompromised mice were obtained through crossbreeding of SOD1/rag2 males and females. Our previous work revealed that SOD1/rag2 mice differ in terms of the number of copies of the mutated SOD1 gene pooling into two groups: high copy number (8 copies) and low copy number (4 copies) [20]. Copy number of the SOD1 gene determines the onset of the disease and the survival period. Therefore, all the mice were genotyped in order to evaluate the effects of transplantation properly.

### 2.5. Q-Cell^®^ Transplantation

Upon thawing, cells were centrifuged at 300× *g,* and the pellet was suspended in saline. Newborn (p2–3) SOD1/rag2 mice pups were cryo-anesthetized and placed in the stereotaxic device equipped with a mouse adaptor. Mice were covered with ice throughout the whole procedure to maintain a low body temperature. 400,000 cells in 4 µL of saline were injected in two separate doses (2 µL each) into each hemisphere using the following coordinates: AP: 0.6, ML: 1.0/−1.0, and DV:0.8 from bregma. After transplantation, mouse body temperature was recovered, and once respiratory function regained normal, the pups were returned to their mothers.

### 2.6. Experimental Design, Tissue and Data Collection

Tissue was collected from 65 mice (26 SOD1/rag2 transplanted, 30 SOD1/rag2 nontransplanted, and 9 rag2 nontransplanted), with additional 11 unique mice (8 SOD1/rag2 nontransplanted and 3 rag2 nontransplanted) for MRI, and additional 115 mice (11 SOD1/rag2 transplanted, 104 nontransplanted) for the assessment of animal survival in the study, which altogether makes 191 individual animals. Gender distribution within the SOD1/rag2 transplanted groups in the survival study was 7:4 females: males. The SOD1/rag2 mice (*n* = 56) were genotyped at 3 weeks of age and divided into groups depending on the copy number of the mutated gene. Altogether, we transplanted *n* = 37 SOD1/rag2 mice. Out of transplanted animals, there were 27 mice classified as long-living 4-copy mice, whereas 10 mice were short-living with 8 copies of the gene. There were additional 3 rag2 mice without transplantation used as a control for IHC, 4 rag2 for Nissl staining, and 3 rag2 for MRI. Magnetic resonance imaging (MRI) was performed on *n* = 5 transplanted 4-copy mice and *n* = 3 of 8-copy mice. For immunohistochemical analysis, *n* = 11 of transplanted brains of 4-copy mice and *n* = 4 of 8-copy mice were collected. Survival analysis was performed on *n* = 5 of 4 copies of transplanted mice and *n* = 6 of 8 copies of transplanted animals. Gender distribution within the 4-copy and 8-copy SOD1/rag2 transplanted groups was 3:2 and 4:2 females to males, respectively. Nontransplanted 4-copy mice constituted *n* = 74 animals (the whole colony was used for survival analysis), whereas 8-copy nontransplanted animals were represented by *n* = 30 animals. We performed not only a Kaplan–Meier estimation but also an ANOVA test to determine the statistical significance of the difference in survival, especially between the transplanted and nontransplanted groups. The tissues for Western blot (WB) analysis were collected from *n* = 10 of 4 copies of transplanted animals, *n* = 10 of nontransplanted SOD1/rag2, and *n* = 5 rag2 mice. Group distribution is shown in Table 2.

### 2.7. Magnetic Resonance Imaging

MR imaging was performed using a 7T scanner (BioSpec 70/30 USR, Bruker, Ettlingen, Germany) equipped with a transmit/receive cylindrical radiofrequency coil (8.6 cm inner diameter, Bruker, Ettlingen, Germany) and a mouse brain dedicated receive-only array surface coil (2 × 2 elements, Bruker, Ettlingen, Germany) as we previously described [19]. In brief, the brains of SOD1/rag2 mice were scanned monthly (at the age of 3, 4, and 5 months) to screen for any morphological changes. Mice were anesthetized with isoflurane (Baxter, Deerfield, IL, USA) at a concentration of 4% in oxygen for induction of anesthesia and then 1.5–2% and positioned in the water-heated MR-compatible bed (head-first, prone). Rectal temperature and respiration rate were monitored during the MR examination (SA Instruments, Stony Brook, NY, USA). High resolution T2-weighted anatomical scans covering the whole brain were used for the evaluation of possible anatomical changes (TurboRARE, TR/TEeff = 7000/15 ms, RARE factor = 4, in-plane spatial resolution = 86 × 86 µm, FOV = 22 × 22 mm, 42 slices 0.35 mm thick, no gap, NA = 4, Scan Time = 23 min).

MRI datasets were analyzed with ImageJ. Rectangle regions of interest (ROI) of the same size were selected within the motor nuclei of the brain for both hemispheres. The mean intensity value of ROI from the left and right hemispheres was measured and normalized to the ROI of the neighboring brain area.

### 2.8. Nissl Staining and MN Counts

Nissl staining was used to evaluate MN morphology and count. Frozen cryostat cut 20 µm spinal cord sections were incubated in xylene 3 times for 3 min. Afterward, sections were twice rehydrated in 100% ethanol for 3 min. Then, staining in 0.1% cresyl violet was performed for 10 min. Slides were rinsed in tap water and washed in 70% ethanol. Dehydration in absolute ethanol was performed. After clearance in xylene, the tissues were mounted in DPX mounting medium (Sigma-Aldrich, St. Louis, MO, USA). Tile scans of whole spinal cord sections were performed on Carl Zeiss Axio Cell Observer Z.1 (Carl Zeiss, Jena, Germany). Neurons were counted manually in both dorsal and ventral horns of spinal cords. On each slide, 4 spinal cord sections were mounted. Counts were taken from sections separated from each other by 11 sections.

### 2.9. Immunohistochemistry

The effect of Q-cell^®^ transplantation on an aggregation of misfolded SOD1 (msSOD1) across the brain and spinal cord of SOD1/rag2 mice was studied at different time points. Immunohistochemical staining was performed for human msSOD1, together with HLXB9 (motor neuron and pancreas homeobox 1) for brain and spinal cord neurons, respectively.

For immunohistochemical analysis, mice were sacrificed after deep anesthesia with an intraperitoneal injection of a mixture of 100 mg/kg ketamine and 1 mg/kg medetomidine in sterile saline. Transcardial perfusion with ice-cold phosphate-buffered saline (PBS) was followed by ice-cold 4% paraformaldehyde solution (PFA). Brains and spinal cords were dissected, postfixed overnight in 4% PFA, and cryopreserved in 20% sucrose at 4 °C until fully saturated. Tissues were then frozen at –80 °C and stored for further analysis. Tissues were cryo-cut into 20 µm sections and stored on glass slides. Staining was performed on representative slices of brains from three transplanted 4-copy mice at the age of 150 days, three transplanted 4-copy mice at 200 days, and five transplanted 8-copy mice at the age of 150 days (no 8-copy mice survived 200 days). At least eight slices were analyzed per mouse. Anti-msSOD1 and anti-HLXB9 (for spinal cord sections) staining were performed on representative slices from five transplanted 4-copy mice at the age of 150 days, six transplanted 4-copy mice at 200 days, four transplanted 8-copy mice at the age of 150 days, six nontransplanted 4-copy mice at the age of 150 days, three nontransplanted 4-copy mice at 200 days, four nontransplanted 8-copy mice at 150 days, and three control rag2 mouse. At least eight slices were analyzed per mouse. Sections were air-dried and washed three times with PBS. Next, they were incubated for 1 h at room temperature (RT) in a blocking solution containing 10% goat serum, 5% bovine serum albumin, and 0.25% Triton X-100. Sections were then stained overnight at 4 °C with the following primary antibodies diluted in PBS: mouse anti-HuNu (MAB1281, Merck KGaA, Darmstadt, Germany; 1:200), rabbit anti-NuMa (#3888S, Cell Signalling, Danvers, MA, USA; 1:100), rabbit anti-Ku80 (ab80592, Abcam, Cambridge, UK; 1:200), mouse anti-STEM121 (#Y40410, Takara Bio, Kusatsa, Japan; 1:100), mouse anti-human misfolded SOD1 clone B8H10 (MM-0070-P, MediMabs, Montreal, Canada; 1:200), and rabbit anti-HLXB9 (PA5-23407, Thermo Fisher Scientific, Waltham, MA, USA; 1:200). The next day sections were washed three times in PBS and incubated for 1 h at RT in the dark with the following secondary antibodies diluted in PBS: goat anti-rabbit Alexa Fluor 546 (A11035, Thermo Fisher Scientific, Waltham, MA, USA; 1:500), goat anti-mouse IgG1 Alexa Fluor 488 (A21121, Thermo Fisher Scientific, Waltham, MA, USA; 1:500), and goat anti-mouse IgG2a Alexa Fluor 546 (A-21133, Thermo Fisher Scientific, Waltham, MA, USA; 1:500). Sections were then washed three times with PBS, and nuclei were counterstained with 4′,6-Diamidino-2-Phenylindole (DAPI; D3571, Thermo Fisher Scientific, Waltham, MA, USA; 1 µg/mL) for 5 min at RT, and again washed three times with PBS and embedded with DAKO fluorescent mounting medium (Dako, Jena, Germany). Images of immunostained slices were acquired using a confocal laser scanning microscope (LSM 780, Carl Zeiss, Jena, Germany) and Cell Observer SD (Carl Zeiss, Jena, Germany) using 10× or 20× objectives. ZEN Black (Carl Zeiss, Jena, Germany) software was used for further image analysis. The analysis was performed in the Laboratory of Advanced Microscopy Techniques, Mossakowski Medical Research Institute, PAS.

### 2.10. Western Blot (WB)

Cerebral cortices, cerebella, and spinal cords from 3–5 transplanted and 4–5 nontransplanted 4-copy mice at the age of 150 and 200 days were analyzed using WB. Mice were sacrificed after deep anesthesia with an intraperitoneal injection of a mixture of 100 mg/kg ketamine and 1 mg/kg medetomidine, diluted in sterile saline. Brains and spinal cords were dissected, homogenized, and lysed in RIPA buffer (10 mM Tris–HCl, pH 7.5 containing 150 mM NaCl, 1% Nonidet P40, 0.1% SDS, 1% Triton X-100, PMSF 0.1 mg/mL) and a proteinase and phosphatase inhibitor cocktail (78441, Thermo Fisher Scientific, Waltham, MA, USA, 1:100). Protein concentration in each sample was measured using the Bio-Rad DCTM protein assay kit (5000112, Bio-Rad, Hercules, CA, USA) according to the manufacturer’s protocol. Samples containing 10 μg of total protein fraction were separated by SDS-PAGE and transferred to nitrocellulose membranes. Membranes were then washed in Tris-buffered saline containing Tween-20 (TBS-T) and incubated in a blocking solution (5% nonfat dried milk dissolved in TBS-T) for 1 h at RT on a rocker. Next, membranes were incubated overnight at 4 °C with the following primary antibodies dissolved in TBS-T: mouse anti-human misfolded SOD1 clone B8H10 (MM-0070-P, MediMabs, Montreal, Canada; 1:250) and rabbit anti-actin (PA1-16889, Thermo Fisher Scientific, Waltham, MA, USA; 1:5000). Membranes were then again washed three times in TBS-T and incubated for 1 h with the following secondary antibodies dissolved in 5% nonfat dried milk: goat anti-mouse IgG, Fab-specific (A2304, Sigma-Aldrich, St. Louis, MO, USA; 1:4000), and goat anti-rabbit IgG, Fab-specific (A0545, Sigma-Aldrich, St. Louis, MO, USA; 1:8000). Membranes were then again washed three times in TBS-T and incubated for 5 min in Amersham ECL Western Blotting Detection Reagent (GE Healthcare Life Sciences, Chicago, IL, USA) according to the manufacturer’s protocol. The chemiluminescent reaction was detected by membrane exposition to X-ray Hyperfilm™ ECL film (70487, GE Healthcare Life Sciences, Chicago, IL, USA) and using G:BOX and GeneSys software (Syngene, Frederick, MD, USA). A semi-quantitative evaluation of protein levels detected by Western blotting was performed by computer-assisted densitometric scanning (GelScan Program; LKB Ultrascan XL, Pharmacia LKB, Piscataway, NJ, USA). The band density was normalized to actin. To standardize the bands and thereby allow cross comparison between blots, the same protein fraction from one specific mouse was included in each blot.

### 2.11. Statistical Analysis

Kaplan–Meier estimation with log-rank Mantel–Cox test and log-rank test for trend were performed using Graph Pad Prism 7.04 (San Diego, CA, USA). Additionally, one-way ANOVA was used to verify if there is a statistical difference in survival between groups (Tukey’s post hoc test) as well as to compare the number of neurons in spinal cord gray matter of transplanted and nontransplanted animals and to estimate the statistically significant difference between groups. As a post hoc test, Sidak’s test was used. Western blot results were compared using one-way ANOVA, and Sidak’s tests with *p* < 0.05 were considered statistically significant.

## 3. Results

### 3.1. The molecular Identity of Q Cells

#### 3.1.1. Immunocytochemical Analysis

**Figure 1:**
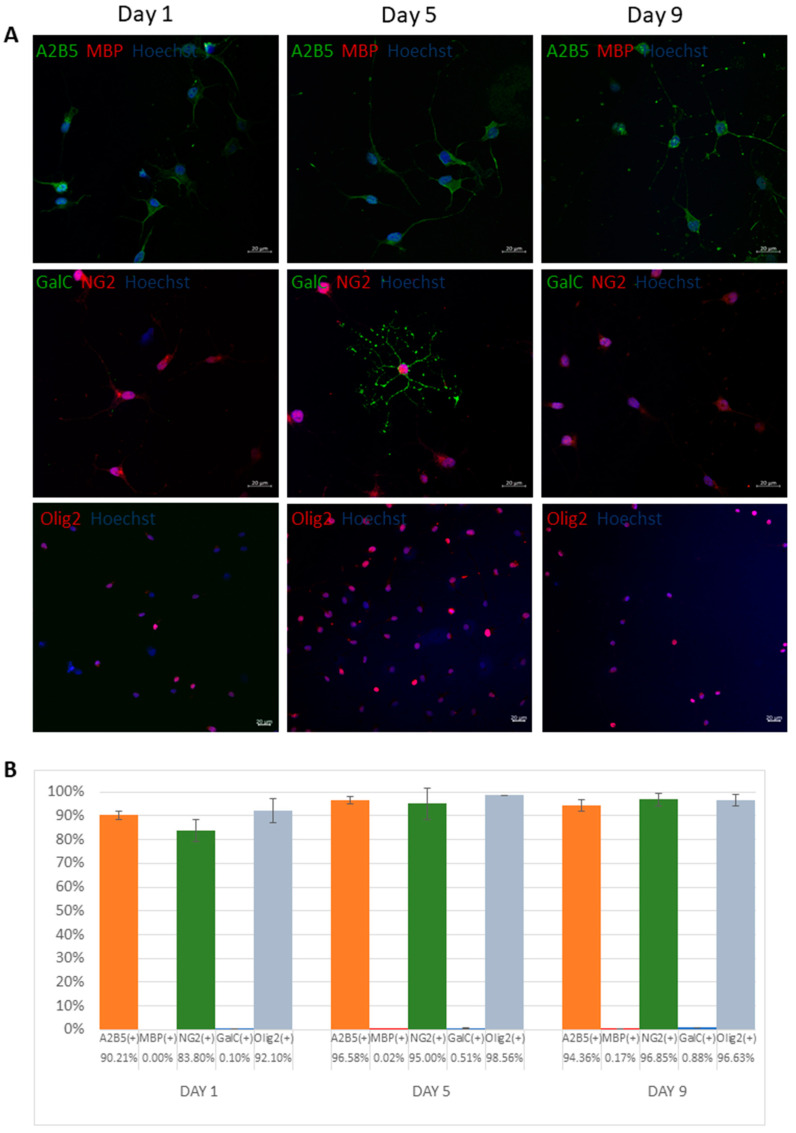
Molecular characteristics of human glial restricted precursors (Q cells). Representative immunocytochemical images based on antibodies anti-A2B5 (green), MBP (red), NG2 (red), GalC (green), and Olig2 (red) (**A**). Percentage of cells (±SD) that express particular antigens (**B**).

GRPs revealed highly positive staining of 90.21, 96.58, and 94.36% and 83.8, 95, and 96.85% on days 1, 5, and 9, respectively, for both glial progenitor markers A2B5 and NG2, respectively (Figure 1A,B). There was also a significant expression of oligodendrocyte precursors marker Olig2 of 92.1, 98.56, and 96.63% on days 1, 5, and 9, respectively. At the same time, there was a scarce expression of mature oligodendrocyte markers such as MBP and GalC (below 1% throughout the experiment).

#### 3.1.2. RT-qPCR Analysis

**Figure 2:**
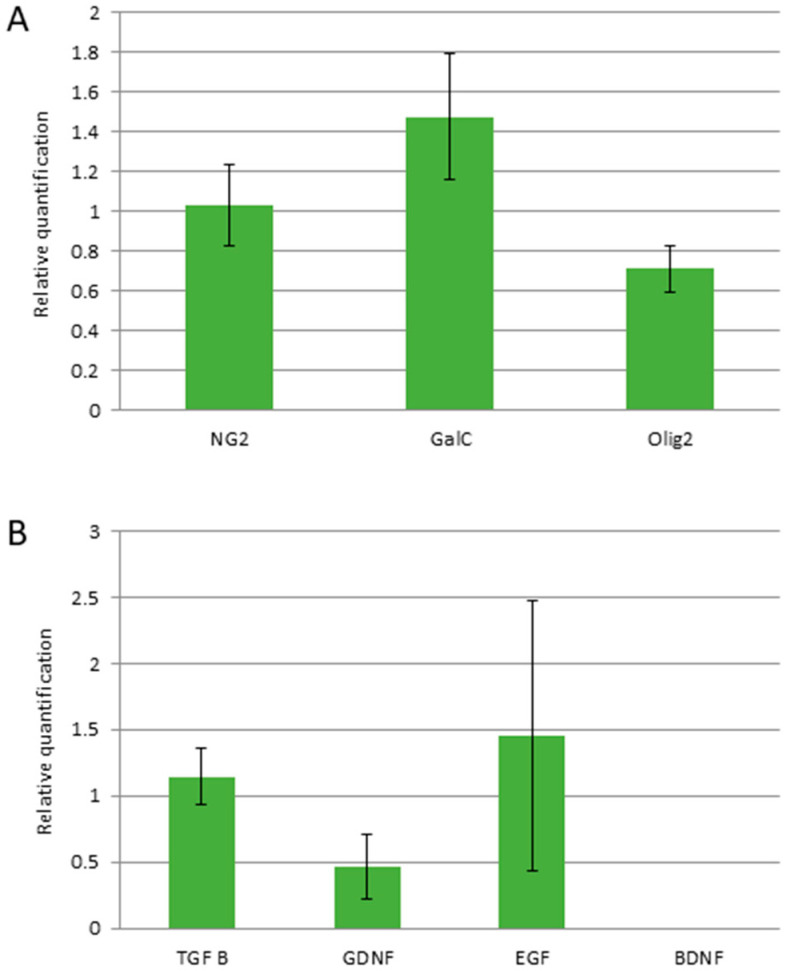
Quantitative analysis of expression of cellular proteins (**A**) and growth factors (**B**) over passages 4–6 (mRNA isolated from 1 mln of cells for each repetition). Values are presented as mean ± SD, *n* = 3.

The RT-qPCR analysis confirmed the relatively stable expression of glial progenitor and oligodendrocyte precursor NG2 and Olig2 genes, respectively, across the observed period until the end of the sixth passage (Figure 2A). There was also found an abundant expression of TGFβ, EGF, and GDNF, while no mRNA for BDNF was detected in Q-cells^®^ over the period of observation (Figure 2B).

### 3.2. Survival Analysis

Control animals with lower copy numbers survived on average 247 (±41) days, whereas 8-copy animals survived 156 (±28) days. The difference between low and high copy number animals was statistically significant (ANOVA with Tukey’s post hoc) *p* < 0.0001. There was no statistical difference between nontransplanted low-copy number animals and their transplanted mates that survived on average 263 (±18) days (*p* = 0.77). Similarly, there was no lifespan prolongation in 8-copy transplanted group that survived 150 (±19) days on average (*p* = 0.98).

**Figure 3:**
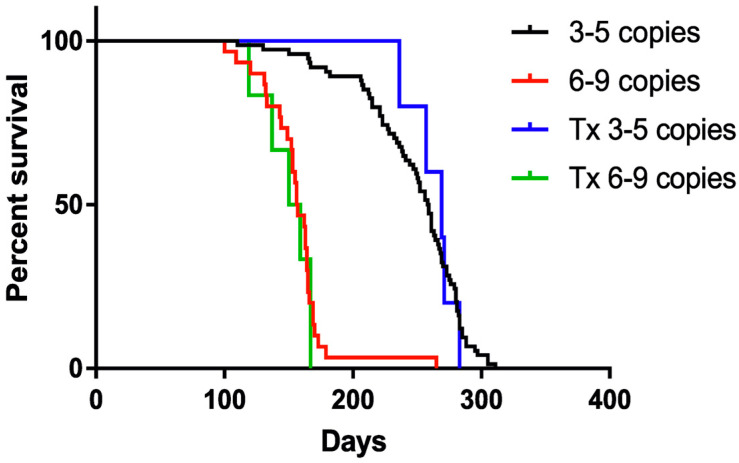
Kaplan–Meier curve presenting the lifespan of transplanted and nontransplanted animals. Sample size within the SOD1/rag2 nontransplanted groups: *n* = 74 (3–5 copies), *n* = 30 (6–9 copies), and within the SOD1/rag2 transplanted groups: *n* = 5 (3–5 copies), *n* = 6 (6–9 copies); *p*-value < 0.0001 (log-rank Mantel–Cox test).

Kaplan–Meier analysis accordingly revealed longer survival of mice with 4 copies of hSOD1 transgene (ca. 250 days) than animals bearing 8 copies (150 days). No prolongation of the life of the animals was observed in any of the transplanted groups (Figure 3), regardless of animal gender. *p*-value < 0.0001 was estimated by log-rank Mantel–Cox test for trend.

### 3.3. MR Imaging of Degenerative Changes in the Brain Stem

**Figure 4:**
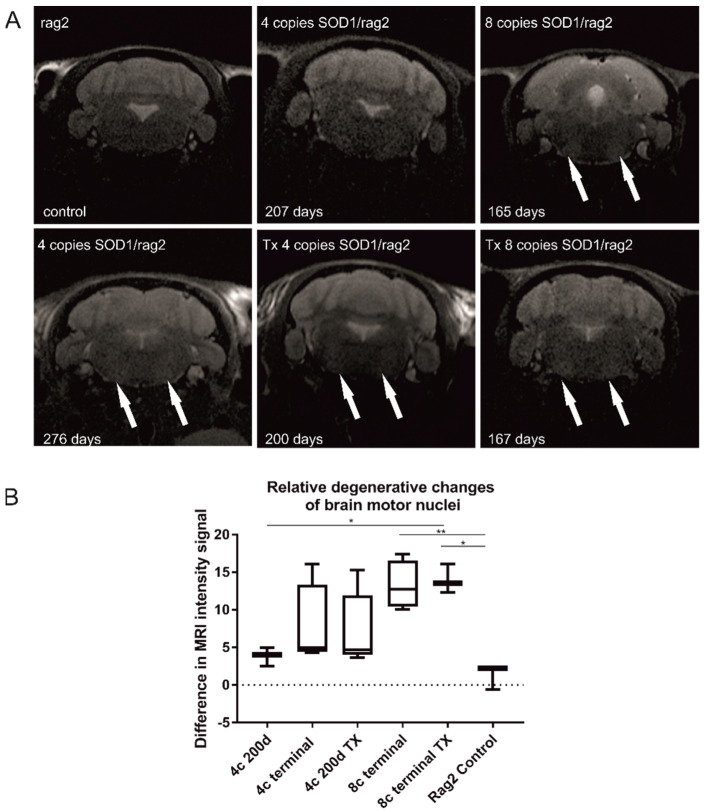
Longitudinal T2-weighted MRI. Hyperintensities within motor nuclei of the medulla are visible, especially in the terminal stage of the disease (165 days old 8-copy animal and 276 days old 4-copy animal, arrows). Neurodegenerative changes are also visible in transplanted animals (Tx 4-copy and Tx 8-copy mice, arrows). The difference in signal intensity between ROI in motor nuclei and ROI localized between motor nuclei—treated as control ROI (**A**). One-way ANOVA test revealed statistical significance: * *p* < 0.05, ** *p* < 0.01 presented on the graph (**B**). Values are presented as mean ± SD. Sample size within the Rag2 control group: *n* = 3; SOD1/rag2 nontransplanted 4-copy group: *n* = 7; SOD1/rag2 transplanted 4-copy group: *n* = 5; SOD1/rag2 nontransplanted 8-copy terminal group: *n* = 5; SOD1/rag2 transplanted 8-copy terminal group: *n* = 3.

We observed hyperintensity in the nuclei of the brainstem that reflects the neurodegenerative changes (Figure 4). They were only visible during the symptomatic stage of the disease in 4-copy animals, while in 8-copy SOD1/rag animals, the changes were detectable earlier, in the presymptomatic period. No difference between 200 days old nontransplanted 4-copy mice and their transplanted counterparts has been found. In the case of 8 copy animals, MR images looked similar for transplanted and nontransplanted animals in the terminal stage, meaning abnormal hyperintensities were visible in motor nuclei for both groups of animals, which suggests unaltered neurodegeneration in this region. The shortcoming of the study is the lack of MRI for the terminal stage in transplanted 4-copy mice (caused by the premature death of this planned group not related to ALS). Degenerative changes in the 4-copy group are less pronounced than in 8-copy animals; therefore, as we do not see the difference between high SOD copy number transplanted and nontransplanted animals, we would not expect differences between transplanted vs. nontransplanted 4-copy mice (Figure 4A). To make the results more transparent, we have measured the difference in hyperintensities in the area of motor nuclei of all imaged animals (Figure 4B). The only significant differences were visible between rag2—control animals and 8-copy nontransplanted mice (*p* < 0.01) and between rag2 and 8-copy transplanted mice (*p* < 0.05). No differences in hyperintensities were visible between transplanted and nontransplanted mice independently of mutated hSOD1 copy number.

### 3.4. Postmortem Analysis

#### 3.4.1. Nissl Staining and Motoneuron Count

**Figure 5:**
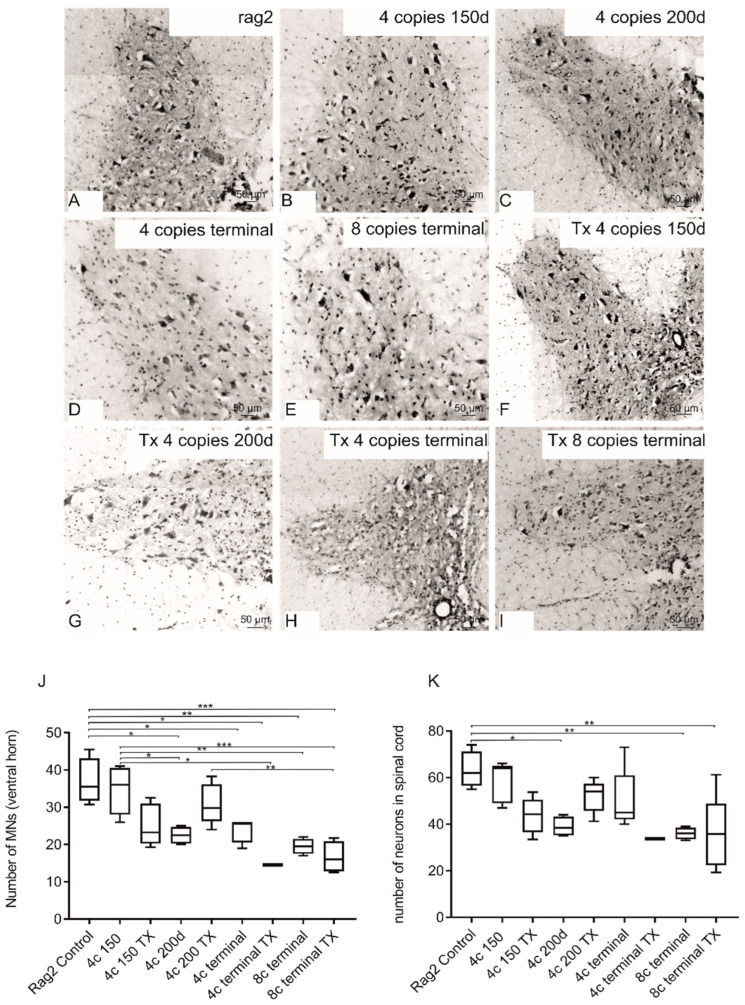
Morphological analysis of MNs in spinal cords of transplanted (**F**–**I**) and nontransplanted animals (**B**–**E**) compared with rag2 control (**A**) show reduced number and size of MNs in SOD1 comparison with rag2 animals. Quantitative analysis (**J**,**K**) did not reveal diminished MN counts in transplanted animals (**J**) as well as in the total number of neurons in the spinal cord (K). The following symbols of the level of statistical significance were adopted: *p* < 0.05 *; *p* < 0.01 **; *p* < 0.001 ***. Values are presented as mean ± SD. Sample size within the Rag2 control group: *n* = 4; SOD1/rag2 nontransplanted 4-copy group at 150 days: *n* = 5; SOD1/rag2 transplanted 4-copy group at 150 days: *n* = 4; SOD1/rag2 nontransplanted 4-copy group at 200 days: *n* = 4; SOD1/rag2 transplanted 4-copy group at 200 days: *n* = 6; SOD1/rag2 nontransplanted 4-copy terminal group: *n* = 5; SOD1/rag2 transplanted 4-copy terminal group: *n* = 1; SOD1/rag2 nontransplanted 8-copy terminal group: *n* = 4; SOD1/rag2 transplanted 8-copy terminal group: *n* = 4.

Nissl staining was performed to assess the morphology and count of MNs (Figure 5). The morphological analysis revealed constricted and pycnotic bodies of MNs, especially in the terminal stage of the disease (Figure 5D,E). Intracerebral transplantation of GRPs did not affect the process of MN degeneration. Both the total number of neurons in whole gray matter and the number of MNs in ventral horns of the spinal cord of transplanted terminal 4-copy animals remained lower in comparison with younger animals as well as control animals (without SOD1 mutation). Similarly, the number of MNs was comparable in nontransplanted and transplanted 8-copy animals. The low number of neuronal cells in animals with cell transplantation was comparable to nontransplanted animals (Figure 5J,K).

#### 3.4.2. Comparison of Localization and Relative Level of Human msSOD1 between Q-Cell-Transplanted and Nontransplanted SOD1/Rag2 Mice

**Figure 6:**
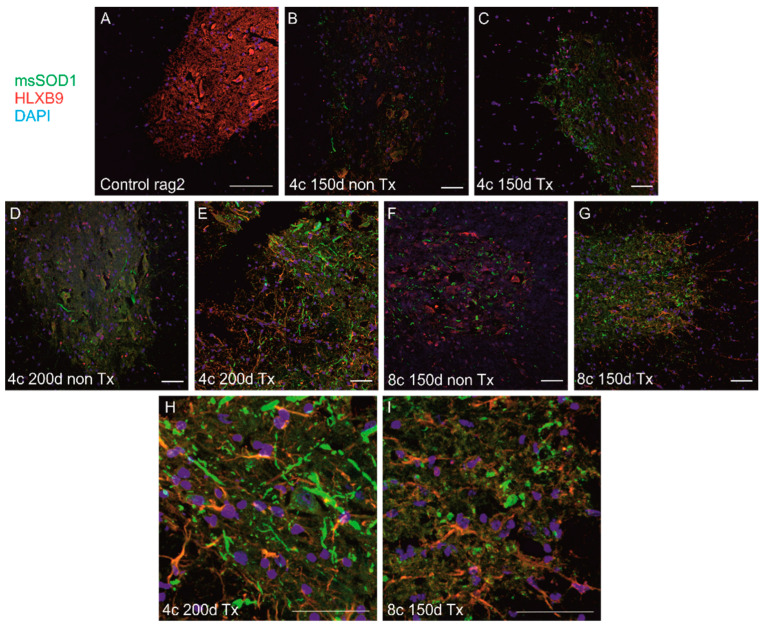
Immunohistochemical analysis of spinal cords from mice transplanted with Q-Cells^®^ and nontransplanted mice. There was no visible difference in spinal msSOD1 aggregation (green) was observed between: nontransplanted (**B**) and transplanted (**C**) 4-copy mice at 150 days; nontransplanted (**D**) and transplanted (**E**,**H**) 4-copy mice at 200 days; nontransplanted (**F**) and transplanted (**G**,**I**) 8-copy mice at 150 days. (**H**,**I**) represent cropped images of (**E**,**G**) respectively. Nontransplanted hSOD1(-) Rag2 mice spinal cord is shown as a control (**A**). HLXB9 was used for neuronal staining (red) and nuclei were counterstained with DAPI (blue). Scale 100 µm. Sample size within the Rag2 control group: *n* = 3; SOD1/rag2 nontransplanted 4-copy group at 150 days: *n* = 7; SOD1/rag2 transplanted 4-copy group at 150 days: *n* = 5; SOD1/rag2 nontransplanted 4-copy group at 200 days: *n* = 3; SOD1/rag2 transplanted 4-copy group at 200 days: *n* = 6; SOD1/rag2 nontransplanted 4-copy terminal group: *n* = 5; SOD1/rag2 nontransplanted 8-copy terminal group: *n* = 4; SOD1/rag2 transplanted 8-copy terminal group: *n* = 4.

**Figure 7:**
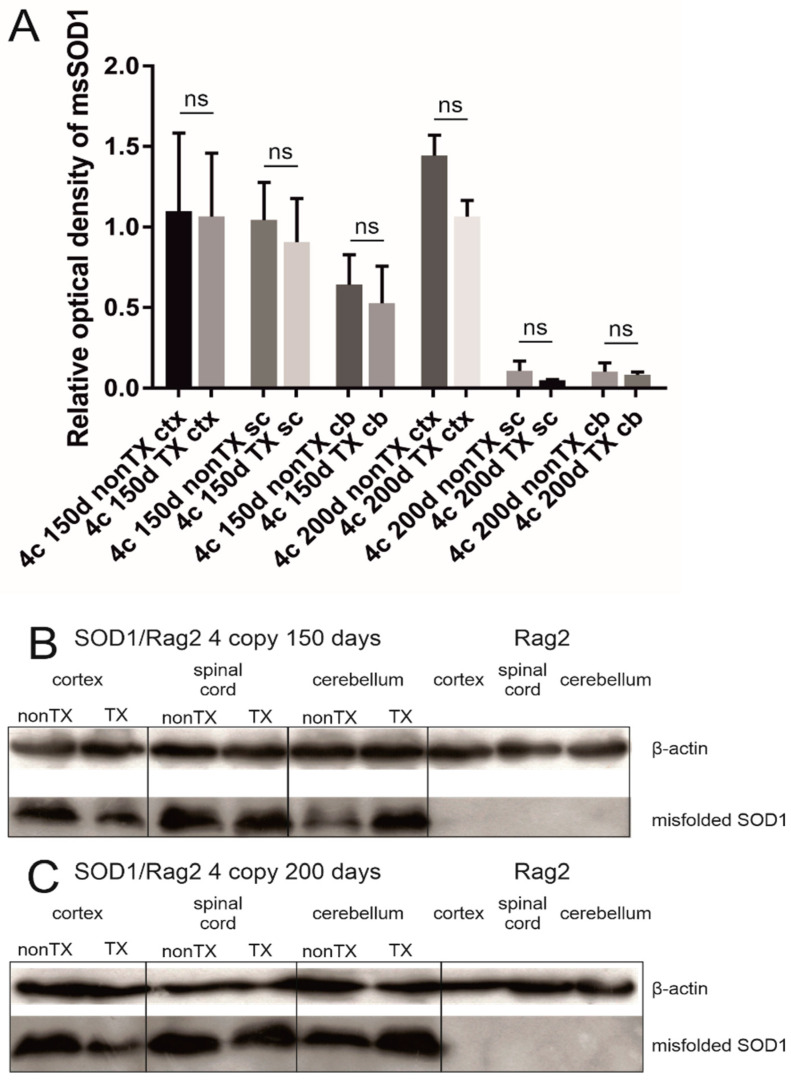
Western blot analysis revealed that there is no significant difference (ns) in the level of expression of misfolded SOD1 between transplanted (TX) and nontransplanted (nonTX) 4-copy hSOD1 animals (150 and 200 days old). Cortices (ctx), spinal cords (sc), and cerebella (cb) were analyzed separately. Rag2 mice are shown as a reference. Results were normalized to actin. The relative optical density of misfolded SOD1 in analyzed tissues (**A**). One-way ANOVA and Sidak’s tests were used with *p* < 0.05 considered statistically significant. Values are presented as mean ± SD. Representative blot from the 150 days mice group (**B**). Representative blot from the 200 days mice group (**C**). Sample size within the Rag2 control group: *n* = 5; SOD1/rag2 nontransplanted 4-copy group at 150 days: *n* = 5; SOD1/rag2 transplanted 4-copy group at 150 days: *n* = 5; SOD1/rag2 nontransplanted 4-copy group at 200 days: *n* = 5; SOD1/rag2 transplanted 4-copy group at 200 days: *n* = 5.

We detected msSOD1 in transgenic animals using IHC and WB, and the level of expression corresponded with the SOD1 copy number and stage of the disease. However, we have not found any difference between transplanted and nontransplanted animals in any compared group at any time point neither using immunohistochemistry nor WBs in the cerebral cortex, cerebellum, and spinal cord (Figure 6 and Figure 7). We identified sizable aggregates of msSOD1 in the spinal cord of both transplanted and nontransplanted animals (Figure 6). We used WB assay to compare human msSOD1 protein levels between transplanted and nontransplanted SOD1/rag2 mice at different time points. We analyzed and compared cerebral cortices, cerebella, and spinal cords from transplanted and nontransplanted 4-copy mice at the age of 150 and 200 days. There were no statistically significant differences between samples from transplanted and nontransplanted animals at any given time point in terms of relative msSOD1 level (Figure 7).

#### 3.4.3. Detection of Human Cells in Transplanted SOD1/Rag2 Mice

In order to investigate the fate of grafted cells, we screened the brains of SOD1/rag2 mice transplanted with Q-Cells^®^ using immunohistochemistry. To identify cells of human origin, we used different antibodies recognizing human antigens: anti-HuNu, anti-NuMa, anti-Ku80 for detection of nuclear proteins, and anti-STEM121 for cytoplasmic protein. We analyzed and compared representative slices of brains from 4-copy mice at the age of 150 days, 4-copy mice at 200 days, and 8-copy mice at the age of 150 days. Despite our efforts, we did not manage to localize any of the mentioned human-specific antigens within any of the transplanted mice (data not shown).

## 4. Discussion

Our extensive study on 191 mice did not reveal positive therapeutic effects of human GRPs transplanted into the cerebral ventricles at the neonatal stage in an immunodeficient model of ALS. It coincided with no long-term survival of transplanted cells, while in our previous study, human GRPs were able to survive over 600 days after transplantation to neonatal mouse brain with no ALS pathology [16]. Notably, there are many publications on smaller groups of animals, which revealed the positive effects of stem cells in ALS [23,24,25,26]. In contrast to these smaller studies, in our previous preclinical study published over ten years ago with a large number of animals (also nearly 200), we have also failed to show any positive effects with stem cells of different origins [18]. Moreover, it has to be emphasized that over that period of more than 10 years, no progress has been made in prolonging the survival of patients with ALS. Recently published early-phase clinical trials with the use of human fetal-derived neural stem cells (hNSCs) [27,28], human umbilical cord mesenchymal stem cells [28], mesenchymal stem cells secreting neurotrophic factors [29], lineage-negative autologous bone marrow-derived cells [30], and adipose-derived stromal vascular fraction [31] reported only modest if any therapeutic effects and benefit has been observed only in relatively small subsets of patients. Therefore, clinical trials in patients with ALS are still in the nascent stages of development [32]. Therefore, there is still a need to continue extensive preclinical cell therapy studies in ALS.

The survival of transplanted cells has been an issue for years. Our previous study revealed an excellent survival of GRPs in immunodeficient, healthy mice. In contrast, transplantation of GRPs to immunocompetent mice with ALS resulted in rapid death of cells, as evidenced by a nearly 95% decrease in bioluminescence over a period of three weeks [33]. In another study, satisfactory survival of allotransplanted mNSCs has been shown in wild-type mice, while a significant drop of viability was observed in ALS mice over the first 4 weeks post-transplantation with complete loss of bioluminescent cells at the endpoint 8 weeks after transplantation, and improvement in motor function was only transient [34]. Being aware of the potentially hostile microenvironment in ALS, we transplanted the cells at the neonatal stage to take advantage of the potentially supportive microenvironment of developing CNS. Since it is technically prohibitive to perform intraspinal injections (the most typical target in the mouse model of ALS), we decided to target cerebral ventricles. This decision was justified by our previous report on an excellent survival of intraventricular transplantation of hGRPs in an immunodeficient mouse model of dysmyelination [16]. We have also developed and characterized an immunodeficient model of ALS with low copy and extended survival [20]. The rationale was that the slower progression of the disease would facilitate engraftment, integration, and acquiring function of hGRPs in the host CNS and, in this way, provide a relatively favorable microenvironment for attaining a function by the transplanted cells. However, no survival of transplanted hGRPs was observed at the study endpoints. It points to the negative impact of the disease progression on the transplanted cells regardless of the immune status of the cell recipient or its developmental stage. Therefore, we conclude that a hostile microenvironment might be a factor limiting the survival of hGRPs, precluding any positive effect and lifespan extension in our study.

However, long-term survival is not always required to exert therapeutic effects. It is particularly evident with sudden onset and inflammation-prone diseases such as stroke [35], traumatic injury [36], or multiple sclerosis [37,38]. However, the previous studies and our projects show that the therapeutic effect of short-lived stem cells does not happen in ALS. Overall, the chronic character of ALS with no sudden cascade activation of the immune system might be behind poor outcomes of short-lived stem cells. Therefore, it seems that a robust survival and integration of transplanted cells are rather necessary conditions to radically impact chronic neurodegenerative disorders such as ALS.

## 5. Conclusions

ALS is in dire need of effective therapies, and stem cells are still an attractive strategy to tackle the disease. However, the body of evidence, including our current study, indicates a premature death of transplanted cells caused by a hostile host microenvironment might be a primary source of failure. The same phenomenon may take place in the clinical setting, but there are currently no robust tools to investigate biodistribution and survival of transplanted cells in patients. Therefore, there is an urgent need to develop cell tracking to better understand cell fates.

## Figures and Tables

**Table 1:** Primers used in real-time PCR analysis.

Gene Name	Primer Sequence
TGFβ (NM_000660)	Forward: GTACCTGAACCCGTGTTGCT Reverse: TAGTGAACCCGTTGATGTCCA
GDNF (NM_000514.4)	Forward: CCAACCCAGAGAATTCCAGA Reverse: AGCCGCTGCAGTACCTAAAA
EGF (NM_001178130.3)	Forward: GACTTGGGAGCCTGAGCAGAA Reverse: CATGCACAAGTGTGACTGGAGGT
BDNF (NM_170734.4)	Forward: CAGGGGCATAGACAAAAG Reverse: CTTCCCCTTTTAATGGTC
Ng2 (NM_001897.5)	Forward: GAGCCCAGGCACGAAAAATG Reverse: GTATGTTTGGCCCCTCCGAA
GalC (NM_001201401.2)	Forward: TCGTTTCCTCAGCCTCATCTC Reverse: CTCCCCTCCTTCCACACATAAG
Olig2 (NM_005806.4)	Forward: CGACTCATCTTTCCTTCTCTAA Reverse: CGCACTTACCTCATCATTG
GAPDH	Forward: ATGGGGAAGGTGAAGGTCG Reverse: GGGGTCATTGATGGCAACAATA

**Table 2:** Animal distribution within experimental groups.

		SOD1/rag2 Tx 4-Copies	SOD1/rag2 Tx 8-Copies	SOD1/rag2 No Tx 4-Copies	SOD1/rag2 No Tx 8-Copies	Rag2 no Tx
Survival		*n* = 5	*n* = 6	*n* = 74	*n* = 30	-
IHC	150 days	*n* = 5	*n* = 4	*n* = 7	*n* = 4 (terminal)	*n* = 3 (126 days)
	200 days	*n* = 6	-	*n* = 3	-	
	>220 days	-	-	*n* = 5	-	
Nissl staining	150 days	*n* = 4	*n* = 4	*n* = 5	*n* = 4 (terminal)	*n* = 4
	200 days	*n* = 6	-	*n* = 4	-	
	>220 days	*n* = 1	-	*n* = 5	-	
WB	150 days	*n* = 5	-	*n* = 5	-	*n* = 5 (28.5 weeks)
	200 days	*n* = 5	-	*n* = 5	-	
MRI		*n* = 5	*n* = 3	*n* = 7	*n* = 5	*n* = 3

## Data Availability

Data is contained within the article.
